# Parenting profiles and dimensions in relation to children’s internalizing and externalizing behaviors in the ECHO program

**DOI:** 10.1371/journal.pone.0356078

**Published:** 2026-09-30

**Authors:** Xiaoye Xu, Lauren C. Shuffrey, Leslie D. Leve, Alexandra D. W. Sullivan, Nissa Towe-Goodman, Margaret M. Swingler, Jean A. Frazier, Isha Jalnapurkar, Sohye Kim, Alexis Hernandez, Amy J. Elliott, Qi Zhao, Daniel P. Keating, Greta N. Wilkening, Chiaying Wei, Cindy O. Trevino, Santiago Morales

**Affiliations:** 1 Department of Psychology, The State University of New York at Cortland, Cortland, New York, United States of America; 2 Department of Child and Adolescent Psychiatry, NYU Grossman School of Medicine, New York, New York, United States of America; 3 Department of Counseling Psychology and Human Services, University of Oregon, Eugene, Oregon, United States of America; 4 Department of Psychiatry and Behavioral Sciences, Center for Health and Community, Weill Neurosciences Institute, University of California, San Francisco, California, United States of America; 5 Frank Porter Graham Child Development Institute, University of North Carolina at Chapel Hill, North Carolina, United States of America; 6 Carolina Population Center, University of North Carolina at Chapel Hill, North Carolina, United States of America; 7 Eunice Kennedy Shriver Center, University of Massachusetts Chan Medical School, Worcester, Massachusetts, United States of America; 8 Department of Psychology, University of Southern California, Los Angeles, California, United States of America; 9 Avera Research Institute, Sioux Falls, South Dakota, United States of America; 10 Department of Pediatrics, University of South Dakota Sanford School of Medicine, Vermillion, South Dakota, United States of America; 11 Department of Preventive Medicine, University of Tennessee Health Science Center, Memphis, Tennessee, United States of America; 12 Department of Psychology and Institute for Social Research, University of Michigan, Ann Arbor, Michigan, United States of America; 13 Department of Pediatrics, University of Colorado School of Medicine, Aurora, Colorado, United States of America; 14 Department of Psychiatry, Columbia University, New York, New York, United States of America; 15 New York State Psychiatric Institute, New York, New York, United States of America; 16 Department of Psychiatry and Behavioral Sciences, University of Washington, Seattle Children’s Research Institute, Seattle, Washington, United States of America; University of Gothenburg, SWEDEN

## Abstract

Parenting behaviors play a critical role in children’s socioemotional development and risk for psychopathology. Prior research has relied on both variable- and person-centered approaches to examine links between parenting and children’s internalizing and externalizing behaviors, yet few studies have integrated these approaches within a single investigation. In the current study, we combined both approaches by first utilizing latent profile analyses to identify profiles of parenting practices across multiple dimensions. We then utilized path analyses to examine whether parenting profiles (person-centered) and individual parenting dimensions (variable-centered) were associated with children’s internalizing and externalizing behaviors. The sample included children participating in the ECHO Cohort (N = 2,752; 49% female; *M*_age_ = 9.90 years, range = 5–18). Three parenting profiles were identified: Higher Positive Parenting–Better Supervision, Lower Positive Parenting–Medium Supervision, and Medium Positive Parenting–Poorer Supervision. The three profiles differed in children’s internalizing and externalizing behaviors, with symptom levels increasing from Higher Positive – Better Supervision to Lower Positive – Medium Supervision, and highest in Medium Positive – Poorer Supervision. At the dimension level, positive parenting was negatively related to both internalizing and externalizing behaviors, whereas inconsistent discipline was positively related to both outcomes. Poor supervision was positively related to externalizing behaviors but was not associated with internalizing behaviors. Together, these findings advance theoretical models of the caregiving environment and its associations with child psychopathology risk, suggesting that interventions should target both specific dimensions and broader profiles of parenting practices.

## Introduction

The links between parenting practices and psychopathology in children (e.g., emotional and behavioral problems) have been demonstrated by many studies (e.g., [1–3]). Most studies have focused on variable-centered analyses examining how individual parenting dimensions predict child outcomes, which provides important information about specific predictors. Person-centered approaches, in contrast, use techniques such as latent profile or latent class analysis to identify subgroups of families based on patterns of multiple parenting behaviors. Although parenting is often conceptualized as a type (e.g., parenting styles), relatively fewer studies have used person-centered analyses to characterize heterogeneous clusters of parenting and link them with child adjustment [4,5].

Although both variable- and person-centered approaches provide complementary insights, studies that have used both approaches to examine the relation between parenting behaviors and children’s mental health outcomes are rare, and such work has often focused narrowly on externalizing problems in relatively small European samples (N < 1000) [6,7]. We addressed these gaps by using both approaches to examine how parenting dimensions (i.e., positive parenting, inconsistent discipline, poor supervision) and parenting profiles defined by these dimensions are related to internalizing and externalizing behaviors in a large, diverse sample in the U.S.

### Variable-centered and person-centered approaches to study parenting

Research on parenting and children’s psychological adjustment has utilized both variable-centered and person-centered analytic approaches. Variable-centered methods, such as regression and structural equation models, examine how individual parenting dimensions (e.g., positive support, discipline consistency, supervision) are associated with variation in children’s emotional and behavioral outcomes across the full sample. These approaches provide estimates of the strength and direction of associations between specific parenting behaviors and child symptoms, supporting questions such as “Does inconsistent discipline predict higher externalizing problems?” and allowing researchers to control for other variables while comparing effect sizes.

Complementing these dimensional analyses, person-centered approaches such as latent profile analysis (LPA) or latent class analysis identify subgroups of families with distinct combinations of parenting behaviors. Rather than focusing on isolated dimensions, person-centered models cluster individuals based on patterns of multiple behaviors, enabling the study of how real-world combinations of parenting practices coalesce and relate to child outcomes. For example, person-centered research has identified profiles related to parenting styles (i.e., highly authoritative, relaxed authoritative, permissive-focused, and inconsistent parenting styles) and showed that children of parents with highly authoritative profiles exhibit fewer internalizing and externalizing problems compared with all other parenting styles [8].

The value of combining variable- and person-centered approaches has been demonstrated in studies that leverage both methods within the same research design. Van Heel et al. [7] found distinct parenting trajectories (e.g., stable high vs. decreasing support) linked to initial externalizing behavior levels, while variable-centered analyses which quantified dimension-specific effects across the entire sample showed no differences in behavior trajectories. Hukkelberg et al. [6] identified three parenting profiles, with harsh parenting linked to the highest conduct problems, while a variable-centered approach showed that harsh discipline and distress were key predictors. Integrating both variable- and person-centered approaches is particularly important because parenting is inherently multidimensional: families rarely engage in singular behaviors in isolation but instead display constellations of practices that shape children’s emotional and behavioral development. Variable-centered analyses detail how distinct caregiving behaviors relate to outcomes above and beyond each other, while person-centered analyses capture how these behaviors cluster in everyday life and how combinations of practices may confer risk or resilience. Together, they provide a more comprehensive picture of parenting’s influence on child internalizing and externalizing symptoms than either approach alone.

### Associations of parenting with children’s behavioral and emotional problems

Within a dynamic caregiving environment, although child characteristics (e.g., temperament) can predict caregiving behaviors [9,10], parenting remains to be a critical environmental predictor of children’s internalizing and externalizing behaviors [11]. Children’s internalizing behaviors include anxiety, depression, and withdrawal [12]. Anxiety and mood disorders are common during adolescence, with lifetime prevalence rates of 32% and 14%, respectively, found among a large and nationally representative sample of adolescents in the U.S. [13]. Children’s externalizing behaviors include aggression, noncompliance, attentional problems, and oppositional behavior [14]. Using a population-based sample in the U.S., Basten et al. [15] found that 7.3% of children aged 6 years demonstrated externalizing behaviors. Meta-analytic reviews indicate that parental warmth, behavioral control, autonomy granting, and an authoritative parenting style are associated with lower levels of internalizing and externalizing behaviors whereas harsh control, psychological control, and authoritarian, permissive, and neglectful parenting are associated with increased levels of both internalizing and externalizing behaviors [1–3,16,17].

Among different parenting dimensions, three specific parenting dimensions (i.e., positive parenting, inconsistent discipline, poor supervision) warrant careful attention due to their documented relevance for both internalizing and externalizing outcomes. Positive parenting refers to parenting behaviors characterized by warmth, responsiveness, praise, and supportive engagement with the child, which foster secure relationships and healthy emotional development [18]. The secure relationships between parent and child, in turn, provides the emotional foundation for children to develop better self-control and emotional management [19] that could lead to lower levels of aggression, noncompliance, and other externalizing problems (e.g., [20,21]). In addition, parents who regularly praise and encourage their children contribute to children’s stronger self-concepts, which in turn are associated with lower levels of depression and other internalizing symptoms (e.g., [22,23].

Inconsistent discipline, defined by unpredictability and a lack of follow-through in enforcing rules [24], can lead to both internalizing and externalizing behaviors. The unpredictability in inconsistent discipline could undermine a child’s sense of environmental stability. This exposure to uncontrollable situations is a known risk factor that contributes to the development of internalizing symptoms like heightened anxiety [11]. Moreover, this same unpredictability can dysregulate the child’s stress response system, which then impairs their self-regulation, leading to an increase in reactive, disruptive behavior and other externalizing symptoms [25].

Poor supervision, characterized by a lack of awareness of a child’s activities, whereabouts, and companions [26], reflects ineffective behavioral control and engagement in children’s daily lives. Maternal behavioral control has been found to have a curvilinear relation to children’s externalizing behaviors [27]. Poor supervision may increase risk for externalizing behaviors by providing more opportunities for children to engage in problem behaviors unchecked. In addition, poor supervision can undermine the child’s emotional security [28], which is a well-established predictor of internalizing behaviors [29].

Besides empirical evidence on parenting and children’s internalizing and externalizing behaviors using variable-centered approaches, researchers have used person-centered approaches to examine parenting behaviors and their associations with children’s outcomes related to psychopathology. Negative parenting profiles, such as harsh-intrusive, problematic, and punitive parenting, have consistently been linked to higher levels of externalizing behaviors, psychological distress, substance use, and disordered eating from infancy to adolescence [30–35]. In contrast, positive parenting profiles, including child-oriented, supportive, and warm styles, have been associated with fewer externalizing and internalizing symptoms [36,37]. Despite all these findings, fewer studies have isolated profiles defined by positive parenting, inconsistent discipline, and poor supervision and evaluated how these profiles relate to internalizing and externalizing outcomes within large, diverse samples. To address these gaps, the present study combines variable-centered and person-centered approaches, analyzing both specific parenting dimensions and broader caregiving profiles within a large, diverse sample to provide a more nuanced understanding of their associations with children’s internalizing and externalizing behaviors.

### The current study

In the current study, we integrated both person-centered and variable-centered approaches to comprehensively examine relations between parenting practices and children’s emotional and behavioral problems with two primary aims. For the first aim which captures a person-centered perspective, we used Latent Profile Analysis (LPA) to determine whether distinct parenting profiles would emerge based on three domains of parenting practices (i.e., positive parenting, poor supervision, and inconsistent discipline) and if those profiles were related to children’s internalizing and externalizing behaviors. We expected that multiple distinct parenting profiles would emerge based on combinations of positive parenting, inconsistent discipline, and poor supervision. We anticipated that profiles characterized by higher levels of positive parenting and lower levels of inconsistent discipline and poor supervision would be associated with lower internalizing and externalizing behaviors, whereas profiles characterized by lower positive parenting and higher inconsistent discipline and poor supervision would be associated with higher internalizing and externalizing behaviors.

Employing a person-centered approach such as latent profile analysis is feasible and informative with large samples because it can detect meaningful subgroups even when they represent a small proportion of the total sample; profiles that comprise 5% or less of participants can be identified reliably only in sufficiently large datasets. Our sample (N = 2,752) is considerably larger than many prior studies that employed person-centered methods (e.g., studies with N < 1000), enhancing the feasibility of identifying distinct parenting profiles that may be relatively rare. Moreover, by including a diverse, multi-site sample from the U.S., this study enhances the generalizability of findings beyond primarily European samples recruited in some previous research.

The second aim employed a variable-centered approach using path analysis to examine how dimensions of parenting practices relate to children’s internalizing and externalizing behaviors across the full sample. Consistent with prior variable-centered findings [2,3], we hypothesized that positive parenting would be negatively associated with both internalizing and externalizing symptoms, whereas inconsistent discipline and poor supervision would be positively associated with internalizing and externalizing behaviors.

## Materials and methods

### Participants

Data for the current study were collected as part of the National Institutes of Health’s (NIH) Environmental influences on Child Health Outcomes (ECHO) Program. ECHO combines extant data from existing longitudinal studies across the U.S. and collects new data through a common protocol to understand how early environmental exposures impact child health and development [38]. Recruitment of the original ECHO cohorts from which these data were drawn occurred between April 6, 2009, and March 2, 2022. Written informed consent was obtained from parents or legal guardians for participation, and child assent was obtained when developmentally appropriate, in accordance with site-specific Institutional Review Board (IRB) approvals. All study procedures were approved by the respective IRBs at participating institutions. The present study involved secondary analysis of de-identified data obtained through the ECHO Data Analysis Center under approved data use agreements; the authors had no access to information that could identify individual participants during or after data collection. Data were accessed for research purposes on 04/08/2022.

The sample for this study consisted of 2752 children (49% female, *M*_age_ = 9.90 years [range = 5–18 years]; *SD*_*age*_ = 3.56) with data from at least one version of the parent-reported Alabama Parenting Questionnaire (APQ, i.e., the full version or the 9-item APQ-SF version) and the Child Behavior Checklist for Ages 6–18 (CBCL/6–18; [39] for school-age children reported by the same parent. Parents completed the APQ and CBCL/6–18 concurrently (i.e., on the same day or within 3 months). See S1 Fig in S1 File for a flow chart of the inclusion and exclusion of the participants. Data were collected across 15 ECHO sites with participants enrolled from 18 states and one territory of the U.S. (California, Colorado, Connecticut, Florida, Illinois, Maryland, Massachusetts, Michigan, Minnesota, New Jersey, New York, North Carolina, Oregon, Pennsylvania, Puerto Rico, Tennessee, Utah, Virginia, and Washington). The majority of participants were White (59.0%), followed by Black (23.0%), multiracial (12.0%), and Other (6.0%). The majority of participants were non-Latinx (84.3%). The median level of prenatal maternal education was a bachelor’s degree, with the range from less than high school to a master’s degree and above. Parents who participated in the study and completed both surveys were primary caregivers, including 2123 biological mothers, 52 biological fathers, and 577 adoptive mothers.

### Measures

#### Parenting behaviors.

Parents reported their parenting behavior on the APQ using either the full version [40] or the APQ-SF [41]. The APQ-SF consists of nine items. For parents who completed the full version, responses to the nine items in the APQ-SF were utilized in the analysis. Each item was presented with a 5-point endorsement scale: Never, Almost Never, Sometimes, Often, and Always. The APQ-SF was scored on three subscales with three items each: positive parenting (e.g., “You praise your child if he/she behaves well”), inconsistent discipline (e.g., “You threaten to punish your child and then do not actually punish him/her”), and poor supervision (e.g., “Your child is out with friends you don’t know”). An average score was computed for each subscale to create three parenting dimensions. The APQ-SF has been validated by parents of school-age children, with good convergent validity [41]. For internal consistency, Cronbach’s α values are.80,.68, and.55 for positive parenting, inconsistent discipline, and poor supervision, respectively, with the current study sample. These reliability coefficients were similar to the internal consistency reported by the original validation of the APQ-SF (Cronbach’s α values = .79,.72, and.59 for mothers’ positive parenting, inconsistent discipline, and poor supervision, respectively) [41]. The relatively low Cronbach’s α values of inconsistent discipline and poor supervision could be due to the small number of items, with each subscale including only three items. For scales with a small number of items, the psychometric literature recommended relying on the mean inter-item correlation to assess internal consistency rather than Cronbach’s α [42,43]. We calculated the mean inter-item correlation for poor supervision to be .32 (range = .28 −.34) and the mean inter-item correlation for inconsistent discipline to be .41 (range = .36 −.48). Both mean inter-item correlations fall within the optimal range of.15−.50 recommended for short scales [43], indicating adequate internal consistency.

#### Child internalizing and externalizing behaviors.

Parents reported child internalizing and externalizing behaviors using the CBCL/6–18 [39]. Parents rated their child’s behavior on 113 items as Not True (0), Somewhat or Sometimes True (1), and Very True or Often True (2). The CBCL/6–18 has eight total syndrome scales. Three of these syndrome scales are related to internalizing problems: anxious depressed, withdrawn depressed, and somatic complaints. Two syndrome scales are related to externalizing problems: rule-breaking behavior and aggressive behaviors. CBCL/6–18’s test-retest reliability and internal consistency were reported as high across different subscales [39]. Both content validity and criterion validity have been established for the CBCL/6–18 [39]. We used the dimensions of externalizing and internalizing behaviors in the current study. These two dimensions were created by adding the values of all items that belong to each dimension. Because CBCL T-scores are standardized by age and gender, they provide developmentally appropriate indicators of children’s externalizing and internalizing behaviors and allow for direct comparisons across ages and CBCL versions [44]. Given the wide age range of the present sample, T-scores were therefore used in the primary analyses. Notably, the pattern of results was unchanged when raw scores were used.

### Statistical analyses

Latent profile analysis models were employed to identify different combinations of participants using the nine items in the APQ in Mplus (version 8.1) [45]. Different statistical indices were calculated to determine the best-fitting model for the current dataset. Lower Akaike information criterion, Bayesian information criterion, and adjusted Bayesian information criterion values indicated a better fit. Higher entropy values (ranging from zero to one) indicated stronger separation between profiles and more accurate classification [46]. The Lo-Mendell-Rubin adjusted likelihood ratio test (LRT) was used to evaluate whether the specified model fit better than a model with one less class [46]. After selecting profiles, we extracted profile membership, which was used in further analyses.

Path analyses using structural equation models were performed through the *lavaan* package in R [47] to examine the associations between parenting profiles and dimensions with children’s psychopathology symptoms. Our hypotheses were tested in two models. Specifically, parenting profile membership was the predictor and children’s internalizing and externalizing behaviors were the outcomes in Model 1. Because profile membership is a three-category nominal variable, it was parameterized using dummy coding with the Lower Positive – Medium Supervision profile (Profile 2) designated as the reference group. This parameterization generated two dummy-coded contrast variables representing Profile 1 (Higher Positive – Better Supervision) relative to Profile 2 and Profile 3 (Medium Positive – Poorer Supervision) relative to Profile 2. Direct paths were estimated from both dummy-coded contrast variables to both child outcome domains simultaneously while adjusting for covariates. In Model 2, parenting dimensions, including positive parenting, inconsistent discipline, and poor supervision, were the predictors and children’s internalizing and externalizing behaviors were the outcomes. Both models controlled for child age, prenatal maternal education, child sex, child ethnicity (Latinx or not), and child race. We included these covariates because they have been found to co-vary with parenting practices (e.g., [48] and predict children’s internalizing and externalizing behaviors (e.g., [49,50]).

For both latent profile analysis and path analysis models, we used full information maximum likelihood estimation to account for missing data and maximum likelihood estimation with robust standard errors to deal with potential nonnormality of the variables [51]. For latent profile analysis, because we used item-level data of APQ, the amount of missing data has the range of 7 (.25%) – 172 (6.25%) across the nine items of APQ. For path analysis, because we used the subscales of APQ (i.e., positive parenting, inconsistent discipline, and poor supervision), the amount of missing data has the range of 1(.04%) –83(3.02%) across the three subscales. The internalizing and externalizing subscales of CBCL did not have missing data since children needed to have data on both subscales to be included in the study.

## Results

### Parenting profiles

Latent profile analysis models were run with the nine items of the APQ from one profile to four profiles. According to the fit indices presented in Table 1, the three-profile model provided the best fit for the data because it was significantly better in model fit than the two-profile model according to all the LRTs (*p*s < .001) and because the four-profile model was not significantly different from it according to the Vuong-Lo-Mendell-Rubin LRT and Lo-Mendell-Rubin LRT (*p*s > .27). The three-profile model also had a high entropy value (.993). Fig 1A displays the means of all items for the three profiles (see S1 Table in S1 File for all *t*-test results), and Fig 1B displays the matrix of parenting profiles by dimension level. On average, in Profile 1 (Higher Positive – Better Supervision), parents had the highest level of the three types of positive parenting (*M*s = 5.00, 4.88, and 4.72) and the lowest level of poor supervision (*M*s = 1.32, 1.05, and 1.09). In Profile 2 (Lower Positive – Medium Supervision), parents had the lowest levels of the three types of positive parenting (*M*s = 3.92, 4.09, and 3.93) and a medium level of poor supervision (*M*s = 1.38, 1.09, and 1.14). In Profile 3 (Medium Positive – Poorer Supervision), parents had a medium level of the three types of positive parenting (*M*s = 4.23, 4.42, and 4.25) and the highest level of poor supervision (*M*s = 2.76, 3.43, and 2.07). The scores for the three items on inconsistent discipline did not differ across the three profiles (*p*s > .05). Of the 2,752 parents, 1,649 (59.92%) were classified in the Higher Positive – Better Supervision profile, 1,042 (37.86%) in the Lower Positive – Medium Supervision profile, and 61 (2.22%) in the Medium Positive – Poorer Supervision profile. Although the last profile was a small portion of the sample, we considered this group to be large enough to constitute a separate group.

**Table 1:** Comparison of model fit for different profile solutions for parenting behavior items.

Number of profiles	AIC	BIC	Adjusted BIC	Entropy	Vuong-Lo-Mendell-Rubin LRT	Lo-Mendell-Rubin aLRT	Parametric Bootstrapped bLRT
1	68410.55	68517.11	68459.92	N/A	N/A	N/A	N/A
2	64609.16	64774.93	64685.96	.997	*p* = .104	*p* = .126	*p* < .001
3	62523.11	62748.07	62627.33	.993	*p* < .001	*p* < .001	*p* < .001
4	60418.79	60702.96	60550.45	.999	*p* = .276	*p* = .280	*p* < .001

*Note.* AIC = Akaike Information Criterion; BIC = Bayesian Information Criterion; LRT = likelihood ratio test; aLRT = adjusted LRT; bLRT = Bootstrapped LRT.

**Fig 1:**
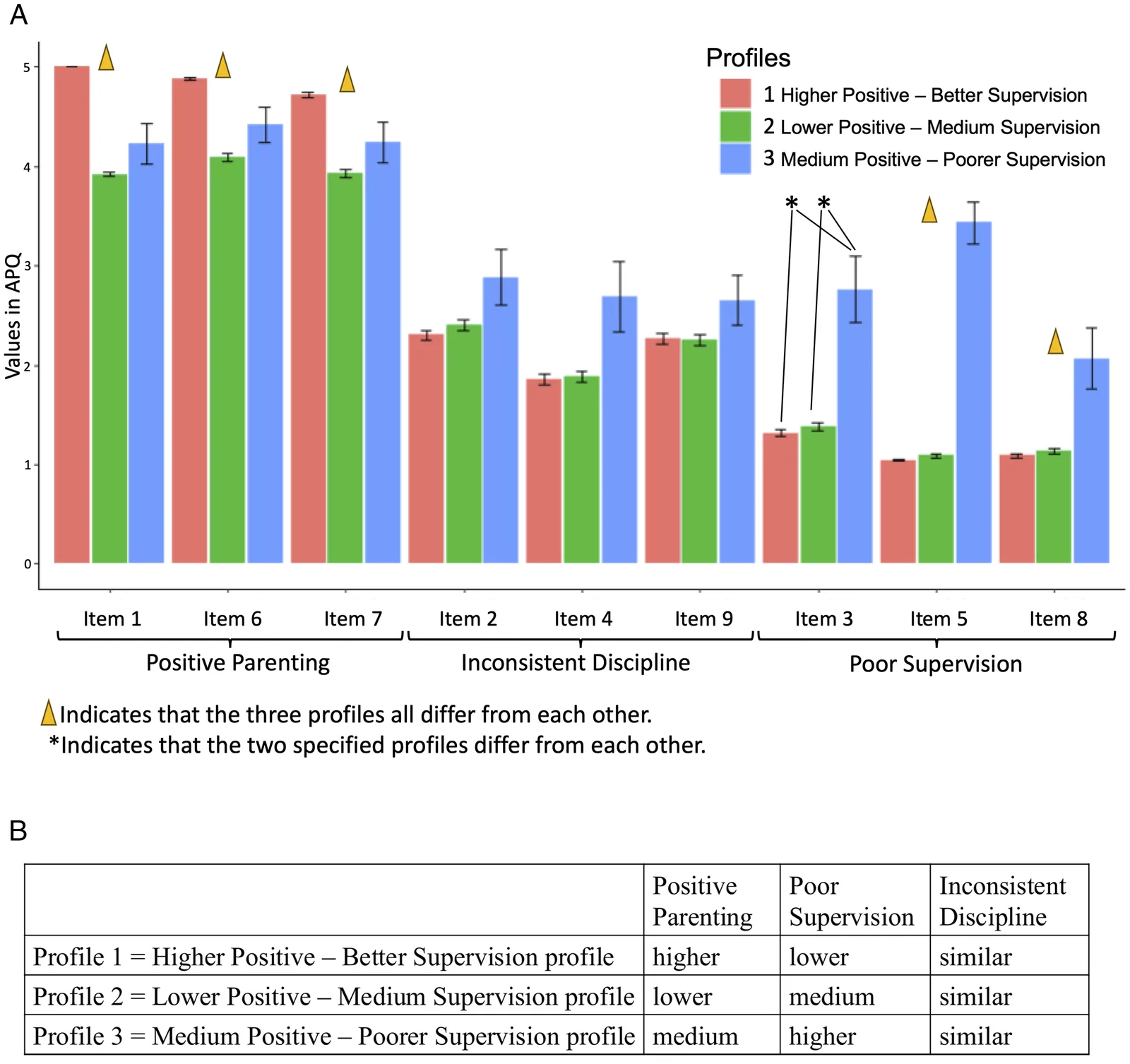
A. The mean of each item in the Alabama Parenting Questionnaire (Short Form) by parenting profiles. The horizontal axis displays the individual items from the Alabama Parenting Questionnaire short form, grouped by subscale. The vertical axis represents the mean score for each item, where higher values indicate a greater frequency of the behavior. Distinct colored bars represent the identified parenting profiles. Error bars represent 95 percent confidence intervals. **B. matrix of parenting profiles by dimension level of Alabama Parenting Questionnaire (APQ).** This matrix maps the intersections between the identified person-centered parenting profiles and their corresponding levels across the individual APQ subscales. This visualization highlights the multi-dimensional caregiving patterns that differentiate each distinct subgroup of parents.

### Descriptive analyses

Descriptive statistics and correlations among all study variables and covariates are presented in Table 2.

**Table 2:** Means, standard deviations, and correlations of all study variables and covariates.

Variable	*M*	*SD*	1	2	3	4	5	6	7	8	9	10	11	12
1. Positive parenting	4.52	.55												
2. Inconsistent discipline	2.17	.73	.00											
3. Poor supervision	1.21	.42	−.12**	.18**										
4. Internalizing problems	48.17	10.27	−.09**	.11**	.07**									
5. Externalizing problems	46.93	10.05	−.08**	.24**	.10**	.54**								
6. Child sex	1.49	.50	−.03	−.01	−.04	−.04	−.08**							
7. Child ethnicity	.16	.36	.03	.07**	−.02	.02	.02	.00						
8. Race: Black	.23	.42	.09**	.12**	.04	−.11**	−.07*	−.02	−.15**					
9. Race: Multi-racial	.12	.33	.01	.00	−.04	.04	.07*	.01	.14**	−.21**				
10. Race: Other	.04	.20	.03	.01	−.03	−.01	−.05	−.02	.23**	−.11**	−.08**			
11. Maternal education	3.38	1.31	−.11**	−.19**	−.11**	.02	−.05	−.02	−.20**	−.45**	.04	.04**		
12. Child age	9.9	3.57	−.07**	−.05*	.20**	.03	−.23**	.04*	−.05*	.15**	−.17**	−.04**	−.25**	
13. Parenting profile: Profile 1 vs. Profile 2	.39	.49	−.79**	.02	.08**	.10**	.10**	.02	−.03	−.16**	.00	−.04	.21**	.00
14. Parenting profile: Profile 1 vs. Profile 3	.04	.19	−.35**	.14**	.66**	.11**	.11**	−.03	−.03	.05*	−.01	−.03	−.02	.09**
15. Parenting profile: Profile 2 vs. Profile 3	.06	.23	.16**	.19**	.68**	.09**	.09**	−.04	−.03	.16**	−.01	−.02	−.12**	.11**

*Note*. *M* = mean; *SD* = standard deviation. For child sex, 1 = male, 2 = female; for child ethnicity, 0 = Non-Latinx, 1 = Latinx; for Black, 0 = Non-Black, 1 = Black; for multi-racial, 0 = Non-multi-racial, 1 = multi-racial; for Other, 0 = White, Black, or multi-racial, 1 = all other races besides not White, Black, or multi-racial; for parenting profile, Profile 1 = Higher Positive – Better Supervision profile, Profile 2 = Lower Positive – Medium Supervision profile, Profile 3 = Medium Positive – Poorer Supervision profile. For Profile 1 vs. Profile 2, 0 = Profile 1, 1 = Profile 2; For Profile 1 vs. Profile 3, 0 = Profile 1, 1 = Profile 3; For Profile 2 vs. Profile 3, 0 = Profile 2, 1 = Profile 3.

*p < .05. **p < .01.

#### Relations of parenting profiles and dimensions and children’s internalizing and externalizing behaviors.

We used two path analysis models to examine the relations of parenting profiles and children’s internalizing and externalizing behaviors (Model 1), and relations of parenting dimensions and children’s internalizing and externalizing behaviors (Model 2). Model 1 demonstrated a good fit for the data based on Hu and Bentler’s [52] guidelines, *χ*^2^(17) = 34.30, *p* = .008; CFI = .99; TLI = .98; SRMR = .01; RMSEA = .02; 95% CI [.01,.03]. As shown in Fig 2A and Fig 2B, Model 1 indicated that, relative to the Lower Positive – Medium Supervision profile (Profile 2), children of parents in the Higher Positive – Better Supervision profile (Profile 1) had lower levels of internalizing and externalizing behaviors (*b*s = −1.93 and −2.23, *p*s < .01, respectively), and conversely, children of parents in the Medium Positive – Poorer Supervision profile (Profile 3) had higher levels of internalizing and externalizing behaviors (*b*s = 4.26 and 4.87, *p*s < .01, respectively). The effects of all variables, including the covariates, are summarized in S2 Table in S1 File.

**Fig 2:**
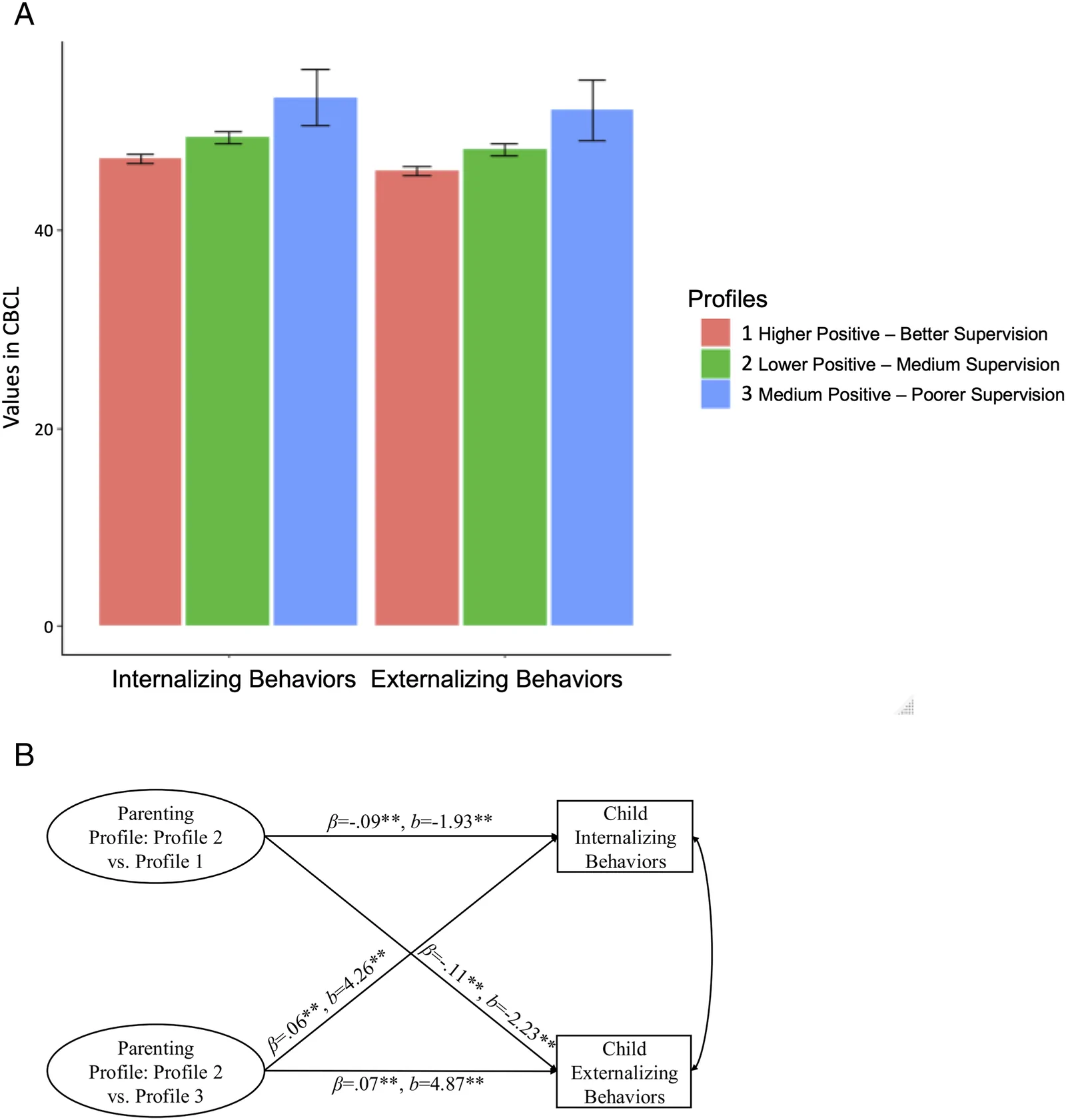
A. The mean of internalizing and externalizing behaviors by parenting profiles. The horizontal axis displays child internalizing and externalizing symptom dimensions, while the vertical axis indicates mean severity scores. Colored bars represent the identified parenting profiles. Error bars reflect 95 percent confidence intervals. **B. Parenting profile are associated with children’s internalizing and externalizing behaviors.** This path model illustrates the relations between the identified parenting profiles and child outcomes. Numerical values along the pathways represent standardized and unstandardized coefficients. Significant paths are in solid lines, and non-significant paths are in dashed lines. To see the full model including nonsignificant paths and covariates with standardized and unstandardized estimates, see S2 Table in S1 File. Parenting profile membership was specified using dummy coding with Profile 2 (Lower Positive – Medium Supervision) serving as the omitted reference group. Path estimates for Profile 1 (Higher Positive – Better Supervision) and Profile 3 (Medium Positive – Poorer Supervision) represent contrasts relative to Profile 2. Prenatal maternal education, child sex, child ethnicity (Latinx or not), child race, and child age were controlled in the model. *p < .05. **p < .01.

The model examining parenting dimensions (Model 2) demonstrated a good fit for the data based on Hu and Bentler’s [52] guidelines, *χ*^2^(19) = 108.37, *p* < .001; CFI = .97; TLI = .90; SRMR = .03; RMSEA = .04; 95% CI [.03,.05]. As shown in Fig 3, Model 2 indicated that positive parenting was negatively related to children’s internalizing and externalizing behaviors (*b*s = −1.38 and −1.57, *p*s < .001, respectively). In contrast, inconsistent discipline was positively related to children’s internalizing and externalizing behaviors (*b*s = 1.61 and 2.88, *p*s < .001, respectively). Poor supervision was positively related to children’s externalizing behaviors (*b* = 2.01, *p* < .001) but was not related to children’s internalizing behaviors (*b* = .90, *p* = .099). The effects of all variables, including the covariates, are summarized in S3 Table in S1 File.

**Fig 3:**
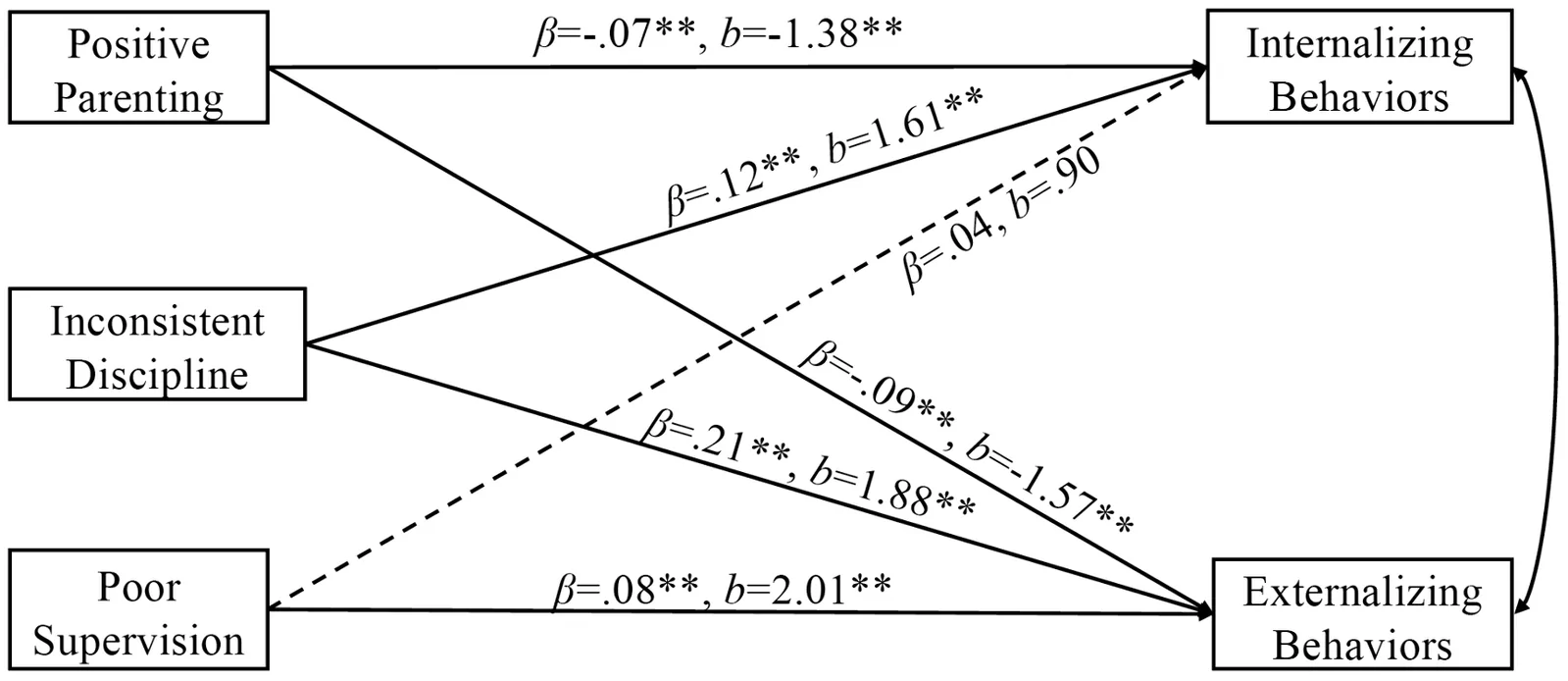
Parenting dimensions are associated with children’s internalizing and externalizing behaviors. This path model illustrates the relations between parenting dimensions and child outcomes. Numerical values along the pathways represent standardized and unstandardized coefficients. Significant paths are in solid lines, and non-significant paths are in dashed lines. To see the full model including nonsignificant paths and covariates with standardized and unstandardized estimates, see S3 Table in S1 File. Prenatal maternal education, child sex, child ethnicity (Latinx or not), child race, and child age were controlled in the model. *p < .05. **p < .01.

For both models, we examined whether demographic variables (i.e., maternal education, child age, and child sex) moderated the associations between parenting profiles or dimensions and children’s internalizing and externalizing behaviors and found no significant moderation effects. Moreover, we conducted a sensitivity analysis controlling for perinatal maternal depression measured through a harmonized scale during pregnancy and infancy [53]. As shown in S4 and S5 Tables in S1 File, the results remained unchanged with similar estimates to the ones presented in the main analyses. Finally, because data of the current study were collected in 15 different ECHO sites, we conducted similar models as Models 1 and 2 using multilevel modeling as sensitivity analyses that accounted for nesting effects within sites. These models were conducted separately for parenting profiles and dimensions. Maternal education was excluded in the model since there was a low number of participants and since multilevel models use listwise deletion. As shown in S6 and S7 Tables in S1 File, the results were the same with similar estimates to the ones presented in the main analyses.

## Discussion

In the current study, we examined whether different parenting profiles and different parenting dimensions were related to children’s behavioral and emotional functioning in a diverse, multi-cohort sample from the U.S. Three profiles of parenting practices were identified: Higher Positive – Better Supervision, Lower Positive – Medium Supervision, and Medium Positive – Poorer Supervision. These profiles differed in children’s internalizing and externalizing behaviors reported by parents, with symptom levels increasing from the Higher Positive – Better Supervision profile to the Lower Positive – Medium Supervision profile, and the highest in the Medium Positive – Poorer Supervision profile. In addition, the findings of a variable-centered approach showed that positive parenting was negatively related to children’s internalizing and externalizing behaviors, while inconsistent discipline was positively related to children’s internalizing and externalizing behaviors and poor supervision was positively related to children’s externalizing behaviors but was not related to internalizing behaviors. Although the sample spanned a wide developmental range, primary analyses used age- and sex-standardized CBCL T-scores to ensure comparability across ages, and the same pattern of findings emerged when raw scores were analyzed, suggesting that results were robust to developmental heterogeneity.

Among all the families who participated in the current study, we found that approximately 60% were characterized by the Higher Positive – Better Supervision profile, approximately 38% were characterized by the Lower Positive – Medium Supervision profile, and approximately 2% were characterized by the Medium Positive – Poorer Supervision profile. The parenting profiles identified in the current study are similar to those identified from items or dimensions of the APQ in previous research. Using the full version of the APQ, Dembo et al. [30] also found that 61% of mothers engaged in parenting behaviors that reflected high involvement and positive parenting, similar to the Higher Positive – Better Supervision profile in the current study. The remaining 39% of the mothers in that study had one of the two profiles: low-level parenting (21%) and a parenting style involving the use of physical punishment (18%) [30]. The distribution of parents across profiles may differ from the current study due to differences between the full and short forms of the APQ, as the short form used in the current study does not include items assessing physical punishment. Both the current study and Dembo et al. [30] found that roughly 40% of parents exhibited patterns of parenting behaviors associated with elevated risk for adverse behavioral and psychological outcomes in youth.

Parenting profiles and parenting dimensions were both related to children’s internalizing and externalizing behaviors. For parenting profiles, the order from lower levels to higher levels of children’s internalizing and externalizing behaviors was Higher Positive Parenting – Better Supervision, Lower Positive Parenting – Medium Supervision, and Medium Positive Parenting – Poorer Supervision. The Higher Positive Parenting–Better Supervision profile reflects a generally more supportive pattern of parenting behaviors, which is consistent with the authoritative parenting style which combines warmth and effective supervision to be the most supportive style among all parenting styles [54]. Thus, as expected, children’s internalizing and externalizing behaviors were the lowest in this profile. As shown in Fig 1A, differences between the two less supportive profiles were more pronounced for poor supervision than for positive parenting, suggesting that poorer supervision may be particularly important for distinguishing children’s emotional and behavioral problems across parenting profiles. This pattern is consistent with prior person-centered studies showing that parenting profiles characterized by less effective or more negative behavioral management (e.g., harsh-intrusive, problematic, and punitive parenting) are associated with higher levels of child maladjustment, including internalizing and externalizing behaviors [30–35].

At the level of parenting dimensions, positive parenting was negatively related to both internalizing and externalizing behaviors, consistent with evidence of associations between responsive, encouraging caregiving and children’s self-concept and emotion regulation, which are in turn related to lower levels of depression, anxiety, aggression, and noncompliance (e.g., [20,22,23]). However, inconsistent discipline, characterized by unpredictability and a lack of follow-through, was positively associated with both internalizing and externalizing behaviors, aligning with theories and data suggesting that unpredictability is related to environmental stability, heightened anxiety, and poorer self-regulation, which in turn are related to higher levels of disruptive behavior [25,55]. Moreover, poor supervision was positively related to externalizing behaviors, consistent with evidence that inadequate monitoring and oversight are associated with higher levels of conduct problems and rule-breaking [56]. In contrast, the nonsignificant relation between poor supervision and children’s internalizing behaviors aligns with prior findings (e.g., [57]), suggesting that poor supervision may be a weaker and less consistent correlate of internalizing symptoms. This relationship may be more distal and operate through indirect pathways. For example, rather than showing a direct relation, poor supervision has been found to be indirectly associated with internalizing behaviors indirectly through children’s self-esteem [58].

In terms of differences between parenting dimensions and profiles, although all three parenting dimensions were related to children’s adjustment, important distinctions emerged across analytic approaches. Person-centered analyses uniquely captured how combinations of parenting behaviors were associated with children’s internalizing and externalizing behaviors. At the profile level, both positive parenting and poor supervision differentiated the three profiles, whereas inconsistent discipline did not. Notably, although poor supervision contributed to meaningful differences across parenting profiles, it was not related to children’s internalizing behaviors when examined as a single dimension in the variable-centered analyses. In contrast, inconsistent discipline was associated with both internalizing and externalizing behaviors at the dimension level but did not differentiate parenting profiles. These findings indicate that variable-centered and person-centered approaches provide complementary information: variable-centered analyses identify specific dimensions linked to children’s adjustment, whereas person-centered analyses reveal how constellations of parenting behaviors jointly relate to children’s behavioral and emotional outcomes. Importantly, the largely consistent pattern of findings across approaches highlights the robustness of the associations between parenting and children’s adjustment.

Importantly, most parents reported high scores on positive parenting and low scores on poor supervision. This finding is in line with previous studies [59] and could reflect high levels of positive parenting and reasonable supervision in the sample. Alternatively, it may be that the observed levels are in part due to desirability phenomena, potentially resulting in overestimated parent self-assessments [60]. Across both variable- and person-centered approaches, the study findings demonstrated that even small differences in self-reported positive parenting and parental monitoring may be important to understand variability in children’s psychopathological symptoms.

It is important to note that parenting effects to children are bidirectional and child characteristics (e.g., temperament) can predict caregiving behaviors [9,10]. Bidirectional associations are also supported by behavioral genetic research, which indicates that children’s genetically influenced traits could shape parenting through evocative gene–environment correlation [61]. Directionality notwithstanding, findings in the current study indicated practical implications for the development of future parenting interventions. The consistent person-centered and variable-centered associations suggest that interventions targeting even modest improvement in positive parenting, consistent discipline, and supervision could meaningfully reduce children’s internalizing and externalizing problems, highlighting that multi-dimensional caregiving could act as a transdiagnostic risk factor which is related to shared vulnerability of general psychopathology. Moreover, multidimensional parenting profiles identified by the current study suggest that interventions may be most effective when adopting a more holistic approach, including broad configurations of parenting practices and specific parenting dimensions. Family-based early intervention programs, home visiting programs, and the nurse-family partnership preventive intervention [62–64] could be well suited to this approach. Specifically, evidence-based interventions utilizing active video-feedback or direct behavioral coaching have robust meta-analytic support for successfully increasing positive, sensitive parenting while correcting inconsistent discipline or poor supervision, such as the Video-feedback Intervention to promote Positive Parenting and Sensitive Discipline (VIPP-SD; [65], Parent-Child Interaction Therapy (PCIT; [66], and the Triple P System [67]. Moreover, because small self-reported parenting differences were meaningfully linked to child adjustment, brief self-report tools could be used for screening and monitoring in resource-limited settings. These brief scales could facilitate risk screening, enabling practitioners to identify distinct caregiving profiles and deliver support to parents who need it.

### Limitations and implications for future studies

Despite utilizing a large and diverse sample in terms of location of data collection, child age, child race and ethnicity, and the reporting caregiver (e.g., biological mother, biological father, or adoptive mother) as well as integrating both variable-centered and person-centered approaches, the limitations of the current study should also be recognized. First, parenting behaviors and children’s outcomes were collected concurrently, so we could not infer the directionality of effects. In the future, researchers should use experimental or longitudinal data to examine the development and the direction of the relationship between parenting profiles and children’s behavioral and emotional outcomes. Second, parenting behaviors and children’s internalizing and externalizing behaviors were reported by the same person. To reduce shared-method variance, researchers could use behavioral tasks or different informants (e.g., parent, teacher, and child) to examine whether the relationship between parenting and children’s adjustment changed without shared-method variance. Third, parenting behaviors were measured by the 9-item APQ-SF [41]. Its brevity limits content coverage and reliability relative to longer measures, and self-reports of parenting could be subject to social desirability bias. This modest measure is a constraint on the data that should be considered when interpreting findings. It underscores the need for future research to incorporate more comprehensive and multi-method parenting assessments to replicate and extend the current findings. Fourth, while we controlled for perinatal maternal depression in sensitivity analyses, we lacked concurrent assessments of parental psychopathology. Because parental psychopathology has been found to be associated with both parenting practices and children’s behavioral and emotional outcomes (e.g., [68,69]), future studies should control for concurrent parental mental health to study the unique prediction from parenting profiles and dimensions.

## Conclusions

This study highlights the value of combining person- and variable-centered approaches to capture the multidimensional nature of parenting. Person-centered analyses identified distinct parenting profiles defined by dimensions of positive parenting, inconsistent discipline, and poor supervision. Variable-centered analyses examined the independent associations of these profiles and dimensions with children’s internalizing and externalizing behaviors. Substantively, findings indicated that both parenting profiles and specific dimensions are meaningfully associated with children’s adjustment in a large, diverse sample. Although the cross-sectional design limits causal inference, the convergence across person- and variable-centered approaches adds to an existing literature on the robust associations between parenting practices and child socioemotional functioning. These findings have practical implications for intervention efforts, suggesting that interventions may be most effective when they target both specific parenting behaviors and broader patterns of parenting practices associated with children’s emotional and behavioral problems.

## Supporting information

S1 File Supplementary figure and tables. This file contains the participant inclusion flowchart and supplementary analytical tables. **S1 Fig.** Flow chart of the inclusion and exclusion of the participants. **S1 Table.** Comparisons of each item in the Alabama Parenting Questionnaire (Short Form) among the three parenting profiles. **S2 Table.** Unstandardized and standardized parameter estimates assessing the relations between parenting profile and children’s psychopathology. **S3 Table.** Unstandardized and standardized parameter estimates assessing the relations between parenting dimensions and children’s psychopathology. **S4 Table.** Relations between parenting profile and children’s psychopathology, controlling for perinatal maternal depression. **S5 Table.** Relations between parenting dimensions and children’s psychopathology, controlling for perinatal maternal depression. **S6 Table.** Relations between parenting profile and children’s psychopathology, controlling for cohort effects. **S7 Table.** Relations between parenting dimensions and children’s psychopathology, controlling for cohort effects.
(PDF)
