# Supplementary material for: Parenting profiles and dimensions in relation to children’s internalizing and externalizing behaviors in the ECHO program
Source: PLoS One. 2026 Sep 30;21(9):e0356078. doi: 10.1371/journal.pone.0356078 (PMC13626372; doi:10.1371/journal.pone.0356078)
Supplement: Supplementary file 1 [file pone.0356078.s001.pdf]

Supporting information

**S1 Fig. Flow chart of the inclusion and exclusion of the participants**	**2**
**S1 Table. Comparisons of each item in the Alabama Parenting Questionnaire (Short Form) among the three parenting profiles**	**3**
**S2 Table. Unstandardized and standardized parameter estimates assessing the relations between parenting profile and children's psychopathology**	**4**
**S3 Table. Unstandardized and standardized parameter estimates assessing the relations between parenting dimensions and children's psychopathology**	**5**
**S4 Table. Relations between parenting profile and children's psychopathology, controlling for perinatal maternal depression**	**6**
**S5 Table. Relations between parenting dimensions and children's psychopathology, controlling for perinatal maternal depression**	**7**
**S6 Table. Relations between parenting profile and children's psychopathology, controlling for cohort effects**	**8**
**S7 Table. Relations between parenting dimensions and children's psychopathology, controlling for cohort effects**	**9**

S1 Fig. Flow chart of the inclusion and exclusion of the participants

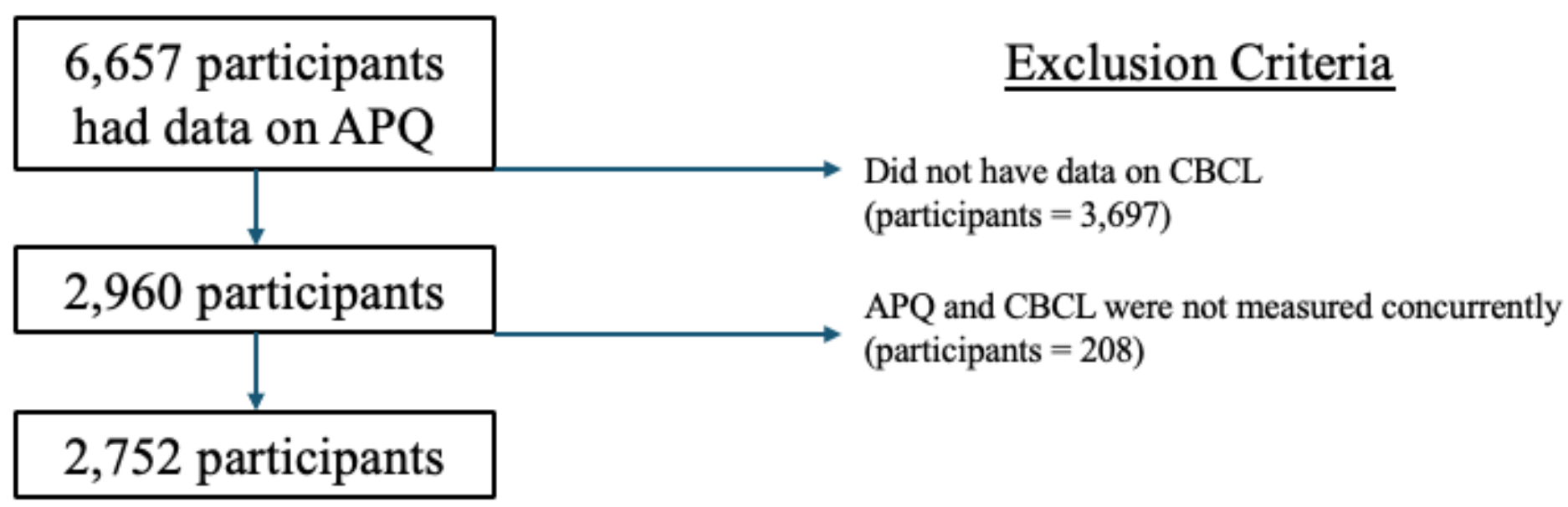

Note. APQ, Alabama Parenting Questionnaire; CBCL, Child Behavior Checklist.

S1 Table. Comparisons of each item in the Alabama Parenting Questionnaire (Short Form) among the three parenting profiles

		Profile 1 (Higher Positive – Better Supervision)			Profile 2 (Lower Positive – Medium Supervision)			Profile 3 (Medium Positive – Poorer Supervision)			
**Item**	**Dimension**	***N***	***M***	***SD***	***N***	***M***	***SD***	***N***	***M***	***SD***	**Significant pairs of profiles**
APQ1	Positive parenting	1644	5.00	.00	1040	3.92	.34	61	4.23	.78	Profile 1-Profile 2**<br>Profile 1-Profile 3**<br>Profile 2-Profile 3**
APQ6	Positive parenting	1642	4.88	.39	1041	4.09	.65	60	4.42	.67	Profile 1-Profile 2**<br>Profile 1-Profile 3**<br>Profile 2-Profile 3**
APQ7	Positive parenting	1637	4.72	.59	1032	3.93	.65	61	4.25	.79	Profile 1-Profile 2**<br>Profile 1-Profile 3**<br>Profile 2-Profile 3**
APQ2	Inconsistent discipline	1645	2.31	.96	1038	2.40	.82	61	2.89	1.10	None
APQ4	Inconsistent discipline	1636	1.86	.99	1041	1.89	.83	61	2.69	1.36	None
APQ9	Inconsistent discipline	1600	2.27	1.00	1017	2.25	.84	60	2.65	.97	None
APQ3	Poor supervision	1524	1.32	.74	997	1.38	.69	59	2.76	1.26	Profile 1-Profile 3**<br>Profile 2-Profile 3**
APQ5	Poor supervision	1538	1.05	.21	999	1.09	.29	60	3.43	.81	Profile 1-Profile 2**<br>Profile 1-Profile 3**<br>Profile 2-Profile 3**
APQ8	Poor supervision	1577	1.09	.40	1010	1.14	.43	61	2.07	1.20	Profile 1-Profile 2**<br>Profile 1-Profile 3**<br>Profile 2-Profile 3**

Note. APQ1–APQ9: all items in the Alabama Parenting Questionnaire (Short Form).
*$p < .05$. **$p < .01$.

S2 Table. Unstandardized and standardized parameter estimates assessing the relations between parenting profile and children's psychopathology

Outcome	Predictors	*β*	*b*	*p*-value
Internalizing problems	Child age	.03	.09	.146
Internalizing problems	Maternal education	-.05	-.40	.074
Internalizing problems	Child sex	-.05	-.94	.015
Internalizing problems	Child ethnicity	.00	.03	.962
Internalizing problems	Race: Black	-.13	-3.04	< .001
Internalizing problems	Race: Multi-racial	.02	.69	.256
Internalizing problems	Race: Other	-.02	-.97	.298
Internalizing problems	Parenting profile: Profile 2 vs. Profile 1	-.09	-1.93	< .001
Internalizing problems	Parenting profile: Profile 2 vs. Profile 3	.06	4.26	.003
Externalizing problems	Child age	-.25	-.72	< .001
Externalizing problems	Maternal education	-.15	-1.14	< .001
Externalizing problems	Child sex	-.07	-1.42	< .001
Externalizing problems	Child ethnicity	-.03	-.70	.246
Externalizing problems	Race: Black	-.10	-2.36	< .001
Externalizing problems	Race: Multi-racial	.01	.19	.765
Externalizing problems	Race: Other	-.06	-2.88	.002
Externalizing problems	Parenting profile: Profile 2 vs. Profile 1	-.11	-2.23	< .001
Externalizing problems	Parenting profile: Profile 2 vs. Profile 3	.07	4.87	.001

Note. For child sex, 1 = male, 2 = female; for child ethnicity, 0 = Non-Latinx, 1 = Latinx; for Black, 0 = Non-Black, 1 = Black; for multi-racial, 0 = Non-multi-racial, 1 = multi-racial; for Other, 0 = White, Black, or multi-racial, 1 = all other races besides not White, Black, or multi-racial; for parenting profile, Profile 1 = Higher Positive – Better Supervision profile, Profile 2 = Lower Positive – Medium Supervision profile, Profile 3 = Medium Positive – Poorer Supervision profile. Profile 2 is the reference group.

S3 Table. Unstandardized and standardized parameter estimates assessing the relations between parenting dimensions and children's psychopathology

Outcome	Predictors	β	b	p-value
Internalizing problems	Child age	.04	.11	.087
Internalizing problems	Maternal education	-.02	-.15	.49
Internalizing problems	Child sex	-.05	-.93	.015
Internalizing problems	Child ethnicity	.00	-.05	.941
Internalizing problems	Race: Black	-.13	-3.18	< .001
Internalizing problems	Race: Multi-racial	.02	.70	.249
Internalizing problems	Race: Other	-.02	-1.09	.246
Internalizing problems	Positive parenting	-.07	-1.38	< .001
Internalizing problems	Inconsistent discipline	.12	1.61	< .001
Internalizing problems	Poor supervision	.04	.90	.099
Externalizing problems	Child age	-.24	-.69	< .001
Externalizing problems	Maternal education	-.09	-.70	.001
Externalizing problems	Child sex	-.07	-1.36	< .001
Externalizing problems	Child ethnicity	-.03	-.77	.197
Externalizing problems	Race: Black	-.11	-2.55	< .001
Externalizing problems	Race: Multi-racial	.01	.20	.738
Externalizing problems	Race: Other	-.06	-3.04	.001
Externalizing problems	Positive parenting	-.09	-1.57	< .001
Externalizing problems	Inconsistent discipline	.21	2.88	< .001
Externalizing problems	Poor supervision	.08	2.01	< .001

Note. For child sex, 1 = male, 2 = female; for child ethnicity, 0 = Non-Latinx, 1 = Latinx; for Black, 0 = Non-Black, 1 = Black; for multi-racial, 0 = Non-multi-racial, 1 = multi-racial; for Other, 0 = White, Black, or multi-racial, 1 = all other races besides not White, Black, or multi-racial.

S4 Table. Relations between parenting profile and children's psychopathology, controlling for perinatal maternal depression

Outcome	Predictors	***β***	***B***	***p*-value**
Internalizing problems	Child age	.17	.26	< .001
Internalizing problems	Maternal education	-.06	-.23	.051
Internalizing problems	Perinatal maternal depression	.19	.13	< .001
Internalizing problems	Child sex	.03	.32	.114
Internalizing problems	Child ethnicity	.01	.18	.593
Internalizing problems	Race: Black	-.11	-1.47	< .001
Internalizing problems	Race: mixed	.01	.13	.671
Internalizing problems	Race: other	-.02	-.64	.171
Internalizing problems	Parenting profile: Profile 1 vs. Profile 2	-.07	-.80	< .001
Internalizing problems	Parenting profile: Profile 1 vs. Profile 3	.06	2.30	.021
Externalizing problems	Child age	-.14	-.23	< .001
Externalizing problems	Maternal education	-.15	-.66	< .001
Externalizing problems	Perinatal maternal depression	.18	.14	< .001
Externalizing problems	Child sex	-.10	-1.19	< .001
Externalizing problems	Child ethnicity	-.02	-.35	.328
Externalizing problems	Race: Black	-.09	-1.18	< .001
Externalizing problems	Race: mixed	.00	.05	.901
Externalizing problems	Race: other	-.05	-1.53	.001
Externalizing problems	Parenting profile: Profile 1 vs. Profile 2	-.08	-.93	< .001
Externalizing problems	Parenting profile: Profile 1 vs. Profile 3	.08	3.18	.001

Note. Externalizing = children's externalizing problems; Internalizing = children's internalizing problems; For child sex, 1 = male, 2 = female; For child ethnicity, 0 = Non-Latinx; 1 = Latinx; For Black, 0 = Non-Black; 1 = Black; For multi-racial, 0 = Non-multi-racial; 1 = multi-racial; For others, 0 = participants who were White, Black, or multi-racial; 1 = all other participants who were not White, Black, or multi-racial; For parenting profile, Profile 1 = Lower Positive – Medium Supervision profile, Profile 2 = Higher Positive – Better Supervision profile, Profile 3 = Medium Positive – Poorer Supervision profile. Profile 1 is the reference group.

S5 Table. Relations between parenting dimensions and children's psychopathology, controlling for perinatal maternal depression

Outcome	Predictors	*β*	*B*	*p*-value
Internalizing problems	Child age	.17	.26	< .001
Internalizing problems	Maternal education	-.03	-.13	.270
Internalizing problems	Perinatal maternal depression	.18	.13	< .001
Internalizing problems	Child sex	.03	.32	.109
Internalizing problems	Child ethnicity	.01	.15	.652
Internalizing problems	Race: Black	-.12	-1.51	< .001
Internalizing problems	Race: mixed	.01	.14	.661
Internalizing problems	Race: other	-.03	-.71	.105
Internalizing problems	Positive parenting	-.07	-.64	.002
Internalizing problems	Inconsistent discipline	.09	.66	< .001
Internalizing problems	Poor supervision	.05	.64	.044
Externalizing problems	Child age	-.14	-.23	< .001
Externalizing problems	Maternal education	-.10	-.45	< .001
Externalizing problems	Perinatal maternal depression	.16	.12	< .001
Externalizing problems	Child sex	-.10	-1.16	< .001
Externalizing problems	Child ethnicity	-.02	-.38	.283
Externalizing problems	Race: Black	-.09	-1.25	< .001
Externalizing problems	Race: mixed	.002	.04	.910
Externalizing problems	Race: other	-.06	-1.63	< .001
Externalizing problems	Positive parenting	-.06	-.67	.001
Externalizing problems	Inconsistent discipline	.17	1.31	< .001
Externalizing problems	Poor supervision	.10	1.31	< .001

Note. Externalizing = children's externalizing problems; Internalizing = children's internalizing problems; For child sex, 1 = male, 2 = female; For child ethnicity, 0 = Non-Latinx; 1 = Latinx; For Black, 0 = Non-Black; 1 = Black; For multi-racial, 0 = Non-multi-racial; 1 = multi-racial; For others, 0 = participants who were White, Black, or multi-racial; 1 = all other participants who were not White, Black, or multi-racial.

S6 Table. Relations between parenting profile and children's psychopathology, controlling for cohort effects

Outcome	Predictors	*b*	*p*-value
Internalizing problems	Child age	.04	.609
Internalizing problems	Child sex	-.85	.029
Internalizing problems	Child ethnicity	-.04	.951
Internalizing problems	Race: Black	-2.44	<.001
Internalizing problems	Race: Multi-racial	.70	.261
Internalizing problems	Race: Other	-1.12	.275
Internalizing problems	Parenting profile: Profile 2 vs. Profile 1	-1.70	<.001
Internalizing problems	Parenting profile: Profile 2 vs. Profile 3	4.76	<.001
Externalizing problems	Child age	-.62	<.001
Externalizing problems	Child sex	-1.24	.001
Externalizing problems	Child ethnicity	.56	.339
Externalizing problems	Race: Black	-.75	.132
Externalizing problems	Race: Multi-racial	.10	.870
Externalizing problems	Race: Other	-2.65	.007
Externalizing problems	Parenting profile: Profile 2 vs. Profile 1	-2.14	<.001
Externalizing problems	Parenting profile: Profile 2 vs. Profile 3	4.67	<.001

Note. N = 2670. Maternal education and parental depression were excluded in the model due to a low number of participants. For child sex, 1 = male, 2 = female; for child ethnicity, 0 = Non-Latinx, 1 = Latinx; for Black, 0 = Non-Black, 1 = Black; for multi-racial, 0 = Non-multi-racial, 1 = multi-racial; for Other, 0 = White, Black, or multi-racial, 1 = all other races besides not White, Black, or multi-racial; for parenting profile, Profile 1 = Higher Positive – Better Supervision profile, Profile 2 = Lower Positive – Medium Supervision profile, Profile 3 = Medium Positive – Poorer Supervision profile. Profile 2 is the reference group.

S7 Table. Relations between parenting dimensions and children's psychopathology, controlling for cohort effects

**Outcome**	**Predictors**	***b***	***p*-value**
Internalizing problems	Child age	.05	.524
Internalizing problems	Child sex	-.84	.034
Internalizing problems	Child ethnicity	-.16	.791
Internalizing problems	Race: Black	-2.86	<.001
Internalizing problems	Race: Mixed	.51	.427
Internalizing problems	Race: Other	-1.35	.199
Internalizing problems	Positive parenting	-1.17	.002
Internalizing problems	Inconsistent discipline	1.69	<.001
Internalizing problems	Poor supervision	.69	.166
Externalizing problems	Child age	-.55	<.001
Externalizing problems	Child sex	-1.25	.001
Externalizing problems	Child ethnicity	.34	.555
Externalizing problems	Race: Black	-1.61	.001
Externalizing problems	Race: Multi-racial	-.06	.922
Externalizing problems	Race: Other	-2.78	.004
Externalizing problems	Positive parenting	-1.52	<.001
Externalizing problems	Inconsistent discipline	3.16	<.001
Externalizing problems	Poor supervision	1.67	<.001

Note. N = 2585. Maternal education and parental depression were excluded in the model due to having a low number of participants. For child sex, 1 = male, 2 = female; for child ethnicity, 0 = Non-Latinx, 1 = Latinx; for Black, 0 = Non-Black, 1 = Black; for multi-racial, 0 = Non-multi-racial, 1 = multi-racial; for Other, 0 = White, Black, or multi-racial, 1 = all other races besides not White, Black, or multi-racial.
